# Computational Analysis Reveals Unique Binding Patterns of Oxygenated and Deoxygenated Myoglobin to the Outer Mitochondrial Membrane

**DOI:** 10.3390/biom13071138

**Published:** 2023-07-17

**Authors:** Andriy Anishkin, Kiran Kumar Adepu, Dipendra Bhandari, Sean H. Adams, Sree V. Chintapalli

**Affiliations:** 1 Department of Biology, University of Maryland, College Park, MD 20742, USA; anisan@gmail.com; 2 Arkansas Children’s Nutrition Center, Little Rock, AR 72202, USA; kkadepu@uams.edu (K.K.A.); bhandarid@archildrens.org (D.B.); 3 Department of Pediatrics, University of Arkansas for Medical Sciences, Little Rock, AR 72205, USA; 4 Department of Surgery, School of Medicine, University of California Davis, Sacramento, CA 95616, USA; shadams@ucdavis.edu; 5 Center for Alimentary and Metabolic Science, University of California Davis, Sacramento, CA 95616, USA

**Keywords:** myoglobin, mitochondria, diffusion

## Abstract

Myoglobin (Mb) interaction with the outer mitochondrial membrane (OMM) promotes oxygen (O_2_) release. However, comprehensive molecular details on specific contact regions of the OMM with oxygenated (oxy-) and deoxygenated (deoxy-)Mb are missing. We used molecular dynamics (MD) simulations to explore the interaction of oxy- and deoxy-Mb with the membrane lipids of the OMM in two lipid compositions: (a) a typical whole membrane on average, and (b) specifically the cardiolipin-enriched cristae region (contact site). Unrestrained relaxations showed that on average, both the oxy- and deoxy-Mb established more stable contacts with the lipids typical of the cristae contact site, then with those of the average OMM. However, in steered detachment simulations, deoxy-Mb clung more tightly to the average OMM, and oxy-Mb strongly preferred the contact sites of the OMM. The MD simulation analysis further indicated that a non-specific binding, mediated by local electrostatic interactions, existed between charged or polar groups of Mb and the membrane, for stable interaction. To the best of our knowledge, this is the first computational study providing the molecular details of the direct Mb–mitochondria interaction that assisted in distinguishing the preferred localization of oxy- and deoxy-Mb on the OMM. Our findings support the existing experimental evidence on Mb–mitochondrial association and shed more insights on Mb-mediated O_2_ transport for cellular bioenergetics.

## 1. Introduction

The continuous delivery of oxygen (O_2_) to skeletal muscles and cardiomyocytes is critical for tissue function. Myoglobin (Mb), an intracellular O_2_-binding heme protein, has traditionally been thought to play a role in tissue O_2_ homeostasis and trafficking, at least in some contexts [1]. For instance, the presence of an exceptionally high Mb concentration in the muscles of many marine birds and marine mammals likely supports their remarkable abilities to dive deep and to hold their breath for long periods of time, as Mb can serve as a significant O_2_ sink [2]. That said, studies in humans have shown that the Mb content in muscles appears stable with exercise training (see, e.g., [3,4]). It has also been suggested that the sole function of Mb in muscle cells is to facilitate O_2_ diffusion through the sarcoplasm of the myocyte and maintain an abundant free concentration of O_2_ at the muscle mitochondria [3]. Unlike tetrameric hemoglobin, which can bind four O_2_ molecules, Mb exists as a monomer, and contains a single binding site for O_2_ [5]. During periods of increased metabolic activity, Mb reversibly binds O_2_, facilitates O_2_ transport from the cell surface (after its delivery by red blood cells) to the mitochondria [6,7,8], and offloads the O_2_ as *p*O_2_ drops, converting oxy-Mb to deoxy-Mb [9,10,11].

Blocking Mb function in pigeon breast and rat cardiac and skeletal muscles using the nitrite of hydroxylamine or phenylhydrazine compounds resulted in a decreased O_2_ uptake, lower work output, and reduced ATP production [12,13,14,15]. In one study, Mb gene knockout (Mb^−/−^) mice showed an accelerated onset of fatigue, and covered a shorter distance, compared to wild-type (WT) mice [16]. However, no performance discrepancies were observed before or after a voluntary wheel-running paradigm in a separate report [17]. Studies on the extensor digitorum longus or soleus muscles from both WT and Mb^−/−^ mice were stimulated ex vivo under normoxic or hypoxic conditions, and the fatigue levels were similar. However, in a normoxic 60 min protocol using a slower frequency of 40 Hz, the muscle preparations from Mb^−/−^ mice exhibited a gradual 12% reduction in soleus force [18,19]. Additionally, there is evidence of the activation of multiple compensatory mechanisms to support O_2_ flux to tissues in Mb^−/−^ mice [18,19,20]. Along with these studies, alternative roles for Mb, beyond O_2_ transport in support of oxidative phosphorylation, have emerged. For instance, Mb binds metabolites such as long-chain fatty acids, long-chain acylcarnitines, lactate, and pyruvate [21,22,23,24,25]. Mb expression is not limited to the skeletal and cardiac muscles. Mb is also expressed in the brown adipose tissue (BAT) [26,27] and, based on studies of gene expression and lipid phenotypes in Mb^−/−^ mouse BAT, we proposed that Mb serves as an O_2_ sensor that participates in pathways that modulate metabolic gene and protein expression [26]. Moreover, based on our recent findings on Mb–lipid interaction, Christen et al. generated a non-lipid-binding Mb mutant (MB_ K45A/F46W/K63A/H64W) from immortalized brown adipocytes, indicating that the majority of the mitochondrial Mb is localized on the OMM, and may be important in enhancing substrate flux along with increasing mitochondrial respiration and thermogenesis [27]. Clanton and others have proposed that when the O_2_ partial pressure (pO2) decreases in muscle or heart tissue, the deoxy-Mb’s ability to produce nitric oxide acts as a regulator for oxidative phosphorylation, by inhibiting the cytochrome c oxidase IV [28,29,30,31,32,33].

The degree to which the traditional and emerging roles for Mb are impacted by their proximity to, and interaction with, mitochondria remain to be explored. In most eukaryotic cells, mitochondria are membrane-bound cell organelles, with outer and inner membranes composed of phospholipid bilayers and proteins. The mitochondrial number and oxidative capacity increase with exercise training, presumably as an adaptive mechanism to support enhanced fuel combustion and adenosine triphosphate (ATP) requirements [34,35,36,37,38,39]. Based on their subcellular localization, morphology, and biochemical properties, two distinct mitochondrial subpopulations have been identified in muscles [40,41,42]. Subsarcolemmal (SS) mitochondria are located underneath the sarcolemma, and are larger in size, whereas intermyofibrillar mitochondria (IMF) are compact structures present between the contractile filaments. Recent studies have shown that the exchange of O_2_ requires an interaction between Mb and mitochondria [43]. When respiring mitochondria were separated from an Mb (oxy-Mb) solution by a semipermeable membrane, there was no evidence of Mb deoxygenation, even at a near-zero O_2_ concentration [44]. Experiments involving natural and artificial bilayer lipids have confirmed that the rate of deoxygenation of Mb in the presence of respiring mitochondria is equal to the rate of O_2_ uptake by mitochondria at pH 7.4. Results have also indicated that positive charges of Mb make non-specific interactions with negatively charged phospholipids in the outer mitochondrial membrane (OMM), with electrostatic forces playing a role in the conversion to deoxy-Mb [43]. Further, fluorescence studies have implicated that Mb does not form any quenching complexes, either with inner mitochondrial membrane (IMM) proteins or with outer membrane proteins [45], thus suggesting the preferential binding of Mb directly to the lipids of the outer mitochondrial membrane. Taken together, these results suggest that the Mb–mitochondrial interaction is an important regulator of the intracellular oxy-Mb/deoxy-Mb pools. Considering the importance of the oxygenation status in lipid, lactate, and pyruvate binding to Mb [21,22,23,24,25], it is intriguing to speculate that Mb–mitochondrial interactions influence the formation and stability of metabolite–Mb complexes.

Given the absence of protein–protein complexes involving Mb in the available databases [46], there is a general lack of understanding with respect to the 3D nature, coordinating Mb residues, and conformational switches of Mb interactions with mitochondria. Evolutionary and structural analysis revealed seven invariant charged amino acid residues (all being Lys, Arg, or His) in the heme vicinity, on the surface of all known Mb proteins [45]. It has been hypothesized that the positively charged amino acid residues of Mb (i.e., Lys, Arg, His) contribute to the Mb–mitochondrial complex formation, with the outer mitochondrial membrane, as the latter is composed of a large fraction of negatively charged phospholipid polar head groups [47]. Coincidentally, these charged amino acid residues of Mb align with the O_2_ entry/exit routes, where the rotation of the distal histidine (His(E7)) makes a short and direct channel between the solvent and heme pocket, for both ligand entry and exit in hemoglobin (Hb) and Mb [48]. While it is known from several experimental studies that Mb directly interacts with the lipids of the outer mitochondrial membrane [27,43,44,45,46,49], the structural underpinning of the process is unknown, and it is still unclear if oxy- and deoxy-Mb would behave differently in this context. In the present study, we assembled and equilibrated the outer mitochondrial membrane, and used molecular dynamics (MD) simulations to study the interaction between Mb and mitochondria, to define the structural determinants and preferred orientation of oxy- and deoxy-Mb toward mitochondria. Our findings support the preferential binding of oxy-Mb to the contact sites of the outer mitochondrial membrane. This binding was mediated by local electrostatic interactions between charged groups of Mb and phospholipids.

## 2. Methods

### 2.1. Construction of Outer Mitochondrial Membrane

The phospholipid composition and topology of the mitochondrial membranes are important factors in the proper functioning of the cell. The lipid composition of mitochondrial membranes is evolutionarily conserved, and is remarkably similar among mammalian species, especially species as close as rats and mice [50,51]. Moreover, while it is well known that the morphology and metabolic activity of mitochondria vary in different organs and subcellular localizations, the overall lipid composition is still very similar across all organs and tissues [50,51]. In contrast, within any single mitochondrion, the composition differs significantly in two opposing monolayers of the membrane, as well as in the regions where cristae contact the outer membrane (referred as “contact sites”) vs. in other locations on mitochondria, and this is true for both the inner and outer membranes [51]. Our primary aim was to ensure the highest level of precision in replicating mitochondrial membranes, which led us to prioritize studies that offered thorough insights into the composition and heterogeneity of these membranes. Instead of relying solely on the limited data specific to mouse skeletal or heart muscle, we sought detailed information that would allow for the reconstruction of a membrane patch suitable for molecular dynamics simulations. For the present study, the lipid composition was based on the combined data for mitochondrial membranes in rodents from publications that provided adequate detail on the composition (the combination of data for rat and mouse livers, with some missing information added from rat skeletal muscle studies). To determine the percentage of phospholipid headgroups, monolayer asymmetry, and compositional bias at the contact sites, we referred to studies that separately analyzed the outer and inner mitochondrial membranes, distinguishing between the contact sites and the average composition of each membrane [50,51,52,53]. However, a recent study on the construction of five different membrane-lipid compositions (MLC) showed that no significant effect on the membrane structural properties, such as the membrane thickness or area per lipid, was detected with the varying MLC [54]. Detailed information regarding the lipid tails of phospholipids, including their length, degree of unsaturation, and other characteristics, was solely accessible for rat skeletal muscle, and was limited to the whole-mitochondria homogenate for each specific lipid headgroup type [52].

**Figure 1:**
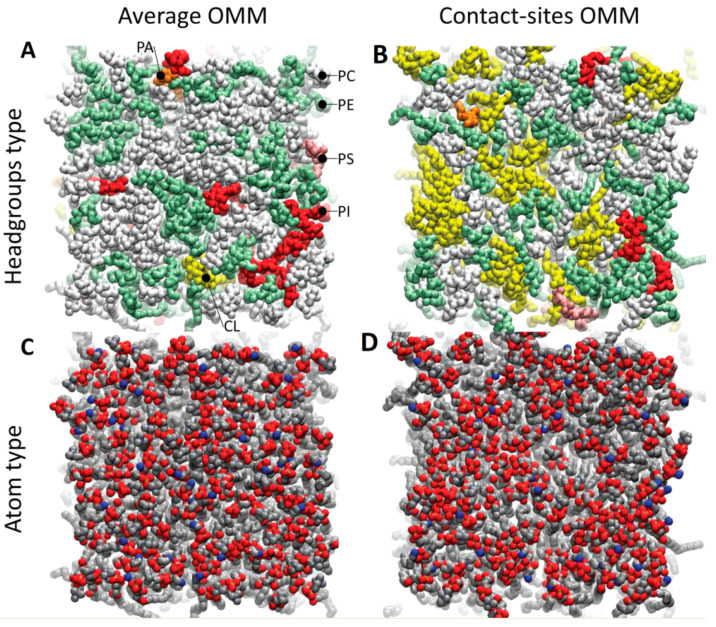
The external surface of the OMM patches after equilibration. The simulations are based on the rat mitochondrial membrane composition, and equilibration continued for 50 (the OMM) or 100 (the CS-OMM) ns, until the areas per lipid and the number of water molecules contacting the bilayer had been stabilized, as described in Methods. (**A**) and (**C**) represents the average composition of the OMM; (**B**) and (**D**) represents the composition of the contact sites of the OMM (the regions where IMM cristae contact the OMM). (**A**) and (**B**) are the membranes colored according to the headgroup type—PC (phosphatidylcholine, white), PE (phosphatidylethanolamine, green), PS (phosphatidylserine, pink), PA (phosphatidic acid, orange), PI (phosphatidylinositol, red), and CL (cardiolipin, yellow). (**C**) and (**D**) are the membranes colored by the atom type—carbon (grey), oxygen (red), nitrogen (blue), and phosphorus (tan) (the last are mostly occluded from view by the oxygens of the phosphate group, in this representation).

To build the lipid patches for our simulations, we first assembled the outer mitochondrial membrane in two variants: one was the “average” outer mitochondrial membrane (OMM), and the other specifically constituted the contact sites of the outer mitochondrial membrane (CS-OMM). The composition of the lipid headgroups and the tails that we utilized is provided in Table 1. Snapshots of the external surface of the outer mitochondrial membrane, illustrating the heterogeneity of the lipid headgroup composition and the abundance of cardiolipin in the CS-OMM, are presented in Figure 1. Our experimental design enabled a focus on Mb–mitochondria associations at the level of phospholipids, as the simulation models excluded typical OMM proteins (the subject of future publications). Our model MLC, in terms of the lipid components (PE, PC, PI, and PS), was nearly in accordance with the model#2 designed by Oliveira et al., in which the OMM-related MD simulations on small-molecule binding and permeation or protein dynamics were accurately reflected, using biochemical studies [54].

**Figure 2:**
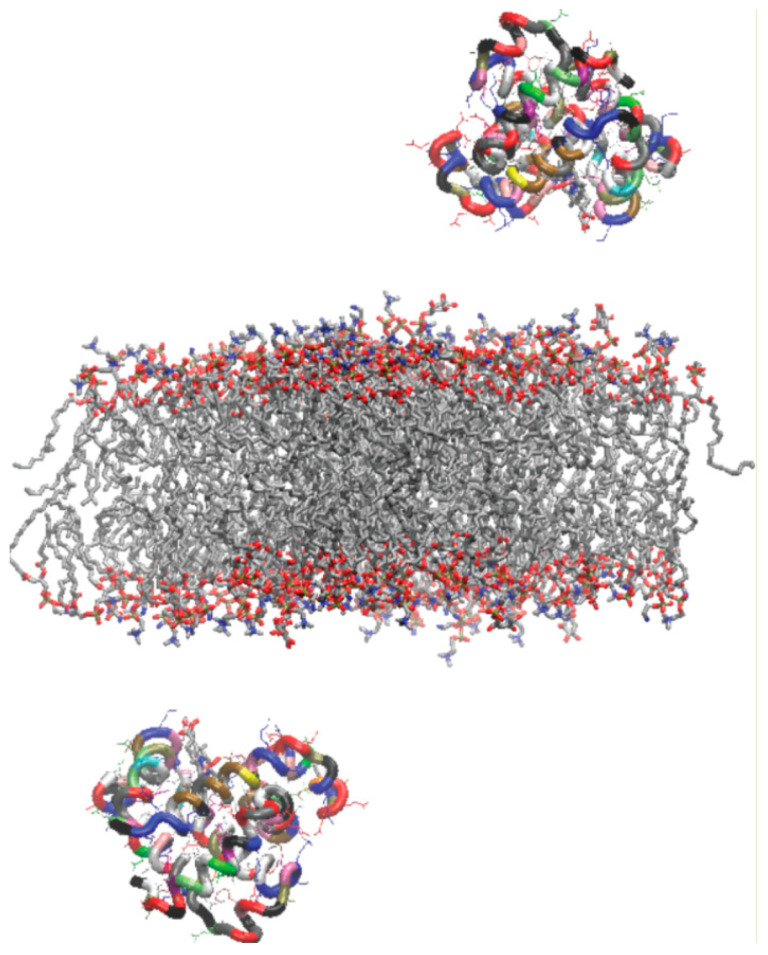
An example of one of six starting arrangements of the simulation system, with a bilayer patch composed of the *external* monolayers of the average outer mitochondrial membrane, and two molecules of oxy-Mb, one on each side, to facilitate the efficiency of the simulations. The lipids are colored according to the atom type (the same as in Figure 1). The Mb protein is illustrated with residues colored according to the amino acid type (GLY, black; ALA, gray; VAL, silver; ILE, LEU, white; PRO, cyan; CYS, yellow; MET, orange; HIS, mauve; PHE, pink; TYR, TRP, purple; ASN, green; GLN, lime; SER, tan; THR, ochre; ASP, GLU, red; ARG, and LYS, blue).

These two membrane patches were constructed using a membrane builder from CHARMM GUI [55,56,57,58], with a 150 mM NaCl solvation box. They were equilibrated for 50 (the OMM) or 100 (the CS-OMM) ns, until the areas per lipid and numbers of water molecules contacting the bilayer were stabilized (see simulation parameters below). The equilibration of the CS-OMM took longer to stabilize, probably because of the higher abundance of cardiolipin, and its tendency to form clusters of a few molecules. In order to enhance the performance of the simulation system, and increase sampling, we replicated the composition of the cytoplasm-facing monolayer from the outer mitochondrial membrane for both monolayers of the simulated membrane patches, namely the OMM and CS-OMM, following equilibration. This allowed us to use two Mbs (one on each site of the membrane) to contact the membrane in the same system simultaneously (see Figure 2 for an illustration of a typical starting structure). These double-outer-monolayer systems were further equilibrated for 10 ns to ensure stability, before Mb was added to the systems.

### 2.2. Oxy- and Deoxy-Mb Models

In this study, the starting mouse Mb structures (oxy- and deoxy-Mb, each complete with a 5 Å hydration shell, along with 3 ions (2 SOD, 1 CLA, sodium and chloride ions, respectively, to make the net charge zero) were taken from our previous published work [25]. In our previous work, the generation of mouse deoxy- and oxy-Mb structures involved the utilization of MODELLER (v9.17) [59,60] through pairwise sequence alignment with horse deoxy-Mb (PDB ID: 2V1K). As horse oxy-Mb was not available, the O_2_ molecule coordinates were instead transferred from sperm whale oxy-Mb (PDB ID: 1MBO) to the deoxy-Mb structure of the horse. Further, to achieve stability and equilibrium, molecular dynamics (MD) simulations were conducted on the resulting oxy-Mb complex for a duration of 10 nanoseconds (ns). Subsequently, the model was employed for docking studies [23,24]. To explore the whole surface of the Mb for possible contacts with the membrane, we used six different orthogonal orientations (like six sides of a cube) of the protein as the starting conformation for the different simulation runs for both oxy- and deoxy-Mb, as displayed in Supplementary Figure S1. In our two-protein system, regardless of the initial orientation, both proteins were orientated in the same manner, with the same side facing the bilayer. However, the lipids present on the membrane surface differed between the two proteins. The two copies of the protein were positioned in such a way as to maximize the lateral distance between them, considering the periodic cell images, maintaining at least 25–30 Å between the closest protein surfaces.

### 2.3. Molecular Dynamics Simulations

For the MD simulations, NAMD2 software [61] was used, and molecular visualizations and simulation analyses were performed using custom-written Tcl scripts in Visual Molecular Dynamics (VMD), a molecular graphics program [62]. All calculations (including the various lipid molecule forcefields specified in Table 1) were carried out using the CHARMM36 forcefield parameters [58,63], with the isothermal–isobaric (NPT) ensemble, TIP3 water model, and periodic boundary conditions [64]. For the O_2_-bound Mb, we used the updated partial atomic charges of the heme prosthetic group and O_2_ molecule from Daigle et al., for which the parameters were calculated and optimized using Ab initio quantum mechanics (QM) [65]. To maintain electroneutrality in each system, adequate amounts of Na^+^ and Cl^−^ ions were added, up to the equivalent of 150 mM salt concentration, to each protein–lipid complex, by replacing the water molecules at random positions in the water box. Throughout the simulation run, a constant pressure (1 atm) and temperature regulation (1 K to 300 K), with a collision frequency of 1.0, were maintained, using Langevin dynamics [66,67]. Both the Van der Waals (VDW) and electrostatic forces were treated using a 12 Å cutoff, with the switching distance 1.5 Å (i.e., the last 1.5 Å before the cutoff distance, where all the non-bonded interactions were linearly tapered down to zero). The long-range electrostatic interactions were treated using the particle mesh Ewald (PME) method [68]. Before the MD simulations, the internal constraints were relaxed via energy minimization for all complexes, and a three-step protocol was employed for the simulation run. In the first step, energy minimization was performed only on the solvent molecules, keeping the protein fixed, using the steepest descent in the first 1000 steps, to avoid clashes between conflicting contacts with the medium. In the second step, we kept the heavy atoms of the oxy-Mb/deoxy-Mb fixed and applied the conjugate gradient method of energy minimization for 1000 steps, while both the solvent and only hydrogen atoms in the oxy-Mb/deoxy-Mb were allowed to relax. In the concluding step, all the solvent molecules and protein atoms were allowed to relax unrestrained for the subsequent 1000 steps during optimization.

The contacts between the non-hydrogen atoms of the lipid, water, and Mb were estimated using a distance cutoff criterion. The distance threshold was assigned separately for each combination of atomic group types (e.g., water O_2_ contacts with the heme non-hydrogen atoms), and it was set such that it would cover the first contact shell. The distance was estimated based on the radial distribution function (the time-averaged probability distribution of the pairwise distances between the atoms of the contacting groups plotted vs. the pairwise distance value). The cutoff distance was defined as the distance of the first minimum of the radial distribution function (i.e., completely covering the most likely position of the atoms in the first contact shell, as represented by the first maximum). At the start of the simulation, the backbone of the oxy-Mb and deoxy-Mb differed only by the root-mean-square deviation (RMSD) of 2.5 Å. At the start of the run, the proteins were positioned with the center of mass 3 nm away from the membrane surface, which would provide at least 4–6 water layers separating the closest protein and lipid regions. The simulation protocol was set as follows:

(1) The slow (40 ns) approach of oxy- or deoxy-Mb toward the membrane, and establishing a stable contact under a constant force (0.01 kcal/mol/Å per atom, the total force 6.13 kcal/mol/Å-on the order of ~1.2 strong H-bonds per Å) uniformly distributed only over the backbone (alpha carbon, and the carbon, nitrogen, and oxygen of the peptide group, 4 atoms per residue in total) of the protein. It allowed the side chains, heme, and O_2_ to adjust freely to the contact. The amount of force used was 11.33 pN per whole protein. These settings were determined empirically, to identify Mb approaching the bilayer and establishing a stable positioning at the lipid surface over the first 20 ns under force (on average) but avoiding a significant distortion in the membrane.
(2) This was followed by 40 ns relaxation without any restraints, to test the stability of contact. The “stability score” of an individual Mb–membrane attachment was quantified as the ratio of (1 + the lowest number of contacts observed) / (the largest number of contacts observed). The contributions of individual protein residues were estimated as the product of (the average frequency of the contacts of the residue with lipids) * (the correlation coefficient between the number of residue–lipid contacts and the “stability score” of the attachment).
(3) The final stage is the 30 ns detachment of oxy- or deoxy-Mb from the membrane surface, using steered molecular dynamics, with the constant velocity of the harmonic restraint, to estimate the force required to break the attachment. The velocity for the detachment (0.5 Å/ns), and the strength of the harmonic restraint for the center of mass (10 kcal/mol/Å^2^), were chosen based on test runs, to be slow enough to minimize the variability of the force between the repetitions, but fast enough to enable a reasonable simulation time. Typically, all contacts between the protein and the membrane were lost within the first 20 ns of steering.

## 3. Results and Discussion

### 3.1. Freedom of Systems to Adjust and Variability of the Contact Arrangement

The outer mitochondrial membrane has a complex and heterogeneous composition, including proteins and a heterogeneous mix of lipids [51,69,70,71,72]. There are two structurally and functionally distinct types of region on the mitochondrial membrane, each with its specific lipid composition: the contact sites (where the inner mitochondrial membrane connects to, or is in closer proximity to, the outer mitochondrial membrane), and the “average” non-contact region [51,52,73,74,75]. As experimental studies of Mb interactions with mitochondria suggest that there is mostly non-specific binding to lipids, rather than to the protein components of the membrane, we focused our simulations on two types of lipid-only patches of the outer mitochondrial membrane: the average composition and the contact-site-specific mixture. However, it should be acknowledged that the proteins in the outer mitochondrial membrane might affect, to a certain extent the structure and dynamics of the nearby lipids and, therefore, bias the pattern and affinity of the Mb binding.

We have used a slow (40 ns) approach to steering the protein toward the mitochondrial membrane. By enabling rotational and translational degrees of freedom for both the protein and the membrane lipids, this step facilitated system adjustment, allowing for the attainment of an energetically favorable arrangement. Over the course of the approach and the establishment of contact, the lipids in the membrane had diffused laterally on a scale of 1–3 nm (Figure S2A). Throughout the process, the Mb exhibited considerable variability in the final arrangement as a result of thermally driven fluctuations, in accordance with the contact establishment protocol. As an example, Figure S2B shows the results of five independent simulations of the Mb–membrane contact arrangements established after 40 ns, starting from an identical starting system, but resulting in translational diffusion in the range of up to 3 nm, and rotation in the range of around 90 degrees in any direction. This indicated a substantial amount of thermal randomization in the system over the course of the simulation, including both the orientation/position of the protein, and the specific lipid composition at the site of the contact. Through the incorporation of six distinct orthogonal orientations of the protein, the initial contact arrangement between the protein and the membrane became essentially random, irrespective of the starting orientation. This randomness implied that the stability of the binding and the energy of detachment will sample a wide range of potential configurations. The RMSD maintained a consistent value throughout all the simulations conducted for both oxy-Mb and deoxy-Mb. This value is comparable to the RMSD of their respective backbones, which arose from thermal fluctuations, and typically stabilized within the initial 15–20 ns.

### 3.2. Statistics of the Initial Contacts between Mb and the Membrane

**Figure 3:**
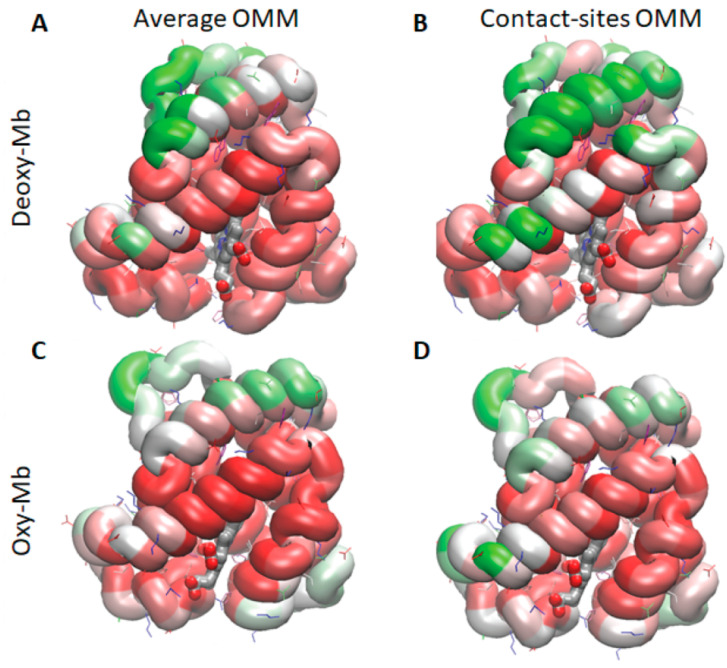
Maps of the initial contact sites of mouse Mb, with the average (**A**) and (**C**) or contact site OMM (**B**) and (**D**) models highlighting the preferential regions of attachment. The frequencies of the contacts between Mb and the OMM lipids were calculated over the whole process of establishing the attachment under constant force, and averaged over all the six simulations per system, with different starting orientations. The Mb protein is colored to denote the lowest (red) to the highest (green) contact frequency. The white color represents zero contribution to stability, and the red and green scales spread to the same absolute values. Most of the residue contacts during this relaxation simulation contributed to the stabilization, rather than the destabilization, of the contacts, resulting in the protein being colored mostly shades of green and white.

While reaching the OMM under a “gentle” constant force, both the oxy-Mb and deoxy-Mb displayed a significant “tumbling” motion, reflecting transient electrostatic interactions with the lipid headgroups, and a steric match between the protein shape and the dynamic lipid surface. However, the time-averaging of the atomic Mb–lipid contact information from multiple runs (six starting orientations of the protein in the bulk for each of oxy-Mb and deoxy-Mb (i.e., twelve simulations in total per system)) revealed clear trends in the contact arrangement, with a preference toward the N-terminal helix and loop regions at the outer “corners” of the protein (Figure 3). While the patterns in the initial contacts are clearly non-uniform around the protein, they are similar for both oxy- and deoxy-Mb, and similar for the contact site OMM and the average-composition OMM. The convergence of four independent sets of simulations suggests that the patterns reflect the common geometric and polar features of the Mb surface, and likely occur at the initial contact with any lipid region in the membrane.

### 3.3. Stability of Myoglobin–Membrane Assemblies in Unrestrained Simulations

To test if the newly established contacts between Mb and the membrane were stable, we first allowed each system to spend 40 ns after the cessation of the external constant force without any restraints. The stability of the attachment mostly fell into two groups that are easy to distinguish: (i) stable (the number of contacts between the membrane and Mb fluctuated around some average value, but did not decrease with time, on average (Figure S3A)), and (ii) unstable (the number of contacts quickly decreased, and sometimes reached zero within the observation time, thus reflecting a loose contact with, or even detachment from, the membrane, by the protein (Figure S3B)). These trends for stable and unstable binding can be illustrated by the snapshots of Mb at the beginning and at the end of 40 ns relaxation (Figure S4). The comparison of the minimum number of membrane–protein atomic contacts remaining after 40 ns relaxation showed that the overall (the average of all the binding orientations) attachment to the non-contact regions of the OMM was less stable than the overall attachment to the contact sites, and this statement is true for both oxy- and deoxy-Mb (Table 2). Table S1 shows a “stability score” (the ratio of the number of contacts at the end of the 40 ns relaxation to the starting number of contacts, quantified as the ratio of (1 + the lowest seen number of contacts)/(the largest seen number of contacts)). The convenience of this quantification was that it allowed distinction between simulations that ended up with the same minimum number of contacts but had very different starting numbers. Therefore, this suggested that the simulations that lost more contacts over the same time frame were less stable, even if they ended up with a similar final number. For both oxy- and deoxy-Mb, the contact site OMM reached a higher stability score, on average, compared to the average OMM.

It should be noted that due to the heterogenous nature of the simulated Mb–OMM arrangements (e.g., the variability between the different regions of Mb in establishing, and being able to maintain, the contact, as well as spontaneous changes in the local lipid composition due to diffusion), our sampling process did not encompass a uniform statistical population; instead, it comprised multiple subsets with varying sites. This understandably led to a large spread in the stability values (from complete contact loss to a stable arrangement), which complicated the statistical comparison. Therefore, the data in the presented tables should be considered as a qualitative reflection of the role of the OMM region and the Mb oxygenation status, while the exact values of the averages might not be very relevant.

### 3.4. Maps of the Residues Contributing to Unrestrained Stability

**Figure 4:**
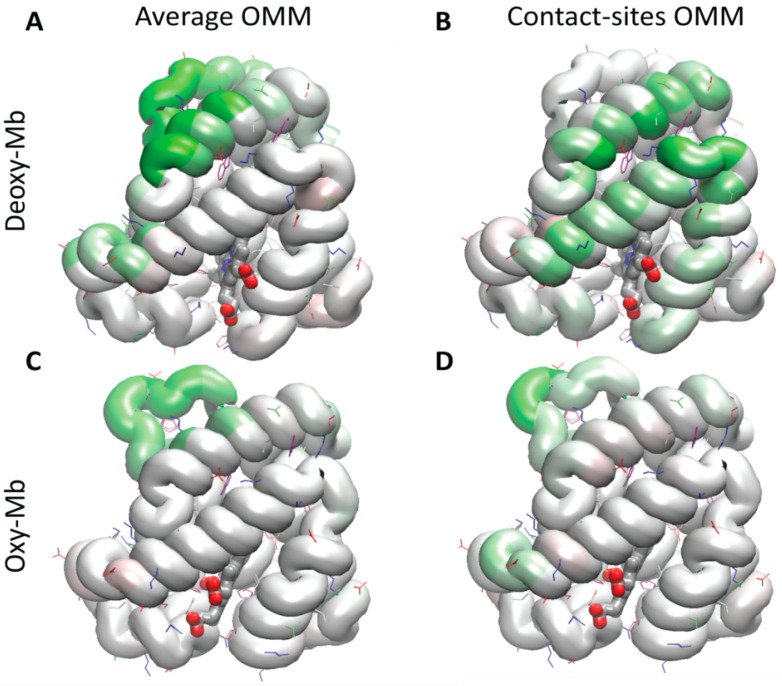
Maps of the stable (unrestrained) contact sites of mouse Mb residues, with the average OMM (**A**) and (**C**) or contact site OMM (**B**) and (**D**) models. The Mb protein is color-coded based on the contributions of the individual residues to the stability of the Mb attachment to the membrane during the 40 ns unrestrained relaxation simulations. The red/pink shade represents the residues that were destabilizing the contact, the white color means essentially no contribution, while the green color shows the regions that contributed the most to stability.

The maps represent the contribution of a particular Mb residues toward the stability of protein–membrane contact during the unrestrained simulations (Figure 4). It was quantified as a product of contact frequency in the time and stability score of attachment described above. The regions of Mb that contributed the most to the stability of the initial contact during the unrestrained simulations were the N-terminal helix and loop regions at the outer “corners” of the protein.

### 3.5. Electrostatic Field of Myoglobin

**Figure 5:**
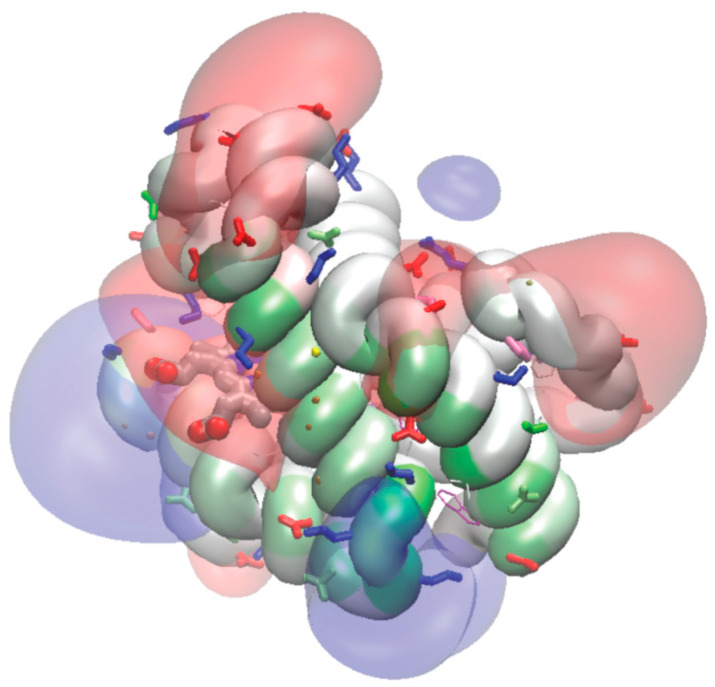
The electrostatic field of mouse deoxy-Mb, in comparison with the map of the residues contributing the most to the stability of the attachment to the contact site OMM. The transparent surfaces indicate the equipotential levels for the positive (blue color) and negative (red) potential at 1000 mV for deoxy-Mb without the medium (i.e., just the protein field). The protein backbone is colored according to the contribution to the attachment stability (green denotes the highest contribution) for the deoxy-Mb at the contact sites of the outer mitochondrial membrane in unrestrained simulations. The spatial relationships between the contacts and the electrostatic field suggest that the most stable regions occur between the positive and negative field maxima, which suggests “opportunistic” local-charge interactions between the protein and transiently exposed lipid polar groups, rather than long-range electrostatic forces.

To assess the long-scale electrostatic interactions of Mb with membrane lipids, and their effect on the preferential orientation and stability of the contact of Mb with the OMM, we calculated the electrostatic field of the protein, and overlapped it over the map of the residues contributing to the stability. The electrostatic field was calculated for Mb in the bulk phase, using particle mesh Ewald’s approximation, and averaged over 40 ns for the two Mb instances. The spatial pattern of protein electrostatics was essentially the same for both the oxy- and deoxy-Mb. As a typical example, Figure 5 shows the distribution of the electrostatic field of deoxy-Mb, in comparison with the contribution of the residues to the unrestrained stability of association with the membrane at the contact sites. Although the regions exhibiting the highest positive or negative potential were commonly found at the “corners” of Mb, both in oxy- and deoxy-Mb, they did not consistently or frequently align with the residues that primarily contributed to the stability of the membrane contact. Instead, the regions of Mb that contributed the most to stability were usually located between the areas with the strongest electrostatic potential. This implies that long-range electrostatic interactions may play a role in influencing the initial contact formation between Mb and the membrane, particularly affecting the protein side facing the lipids. On the other hand, short-range interactions, involving the localized charges of polar groups within the protein and lipids situated between more polar regions, are likely crucial for maintaining stable contact.

### 3.6. Mb–Membrane Contact Strength Test in the Steered Detachment Simulations

To assess the strength of the connections formed between Mb and the outer mitochondrial membrane (OMM), we conducted steered molecular dynamics (MD) simulations. In these simulations, harmonic restraints were applied to the center-of-mass of the protein, gradually moving away from the membrane over a span of 30 ns. Throughout this process, rotational and translational movements were allowed, and the force necessary for a linear increase in the separation distance was monitored. In Table 3, the attachment strength is quantified as the average force required during the separation course. A higher time-averaged amount of force indicates a stronger attachment (highlighted in green in the tables below), whereas the smallest amount of force (highlighted in red) indicates loose or essentially absent contact. The comparison of the results from the average outer membrane patch (left panel) and contact sites (right panel) indicated that the deoxy-Mb binds weaker at contact sites, whereas the oxy-Mb has stronger binding. Interestingly, in all the systems, the average force required for the detachment (32.4–66.7 pN) was 3–6 times higher than the constant force under which the contact was established in the first place (11.33 pN). Although the two simulations employed distinct force application protocols, a direct comparison between them is not feasible. However, it is noteworthy that the timescale and distance at which Mb established or severed contact with the lipids were similar in the two protocols. Furthermore, the significant differences in the forces applied indicated that the interaction between Mb and the membrane was generally favorable.

### 3.7. Contributions of Specific Amino Acid and Lipid Types to the Stability of Mb–Membrane Contact in the Steered Detachment Simulations

Given the pronounced difference between the average forces required for the detachment of deoxy-Mb and oxy-Mb in the OMM vs. the contact-site OMM, we attempted to quantify the types of lipids and amino acid that contributed the most to the attachment-stabilizing interactions for each of the four Mb–membrane combinations. We analyzed the atomic contacts based on the first minimum in the radial distribution function for the Mb–membrane heavy atoms, as described in Methods, and summarized the results for the specific types of amino acids and lipids (Table S2). It is important to acknowledge that the heterogeneous lipid composition of the outer mitochondrial membrane (OMM), and the non-uniform distribution of amino acid types on the regions in contact with Mb, contributed to variations in the correlation estimates between the residue types and detachment forces. Despite the large total length, and the number of independent detachment estimations (up to 12 per Mb–membrane combination), not all residue types yielded sufficient data to accurately assess their correlation with the detachment force. However, the types of residues predicted to have contributed the most can be readily seen from the data.

The analysis indicates that in attachments to the average-composition OMM, both deoxy- and oxy-Mb were interacting mostly with PC and PE lipids. Deoxy-Mb, which required more force in these simulations against the average-composition OMM when compared to oxy-Mb, was displaying on average 30–40% more contacts with these lipids, with PC having a very high correlation (0.65–0.77) with the force required for detachment. From the perspective of the amino acid residues that mediated this contact, for both deoxy- and oxy-Mb, Lys (mostly contacting phosphate oxygens) and Gly were among the most frequent. However, they both showed a very low and, for oxy-Mb, lightly negative correlation with the average force. Considering both the correlations and the number of contacts, for deoxy-Mb, the main stabilizing role could be attributed to acidic residues (Asp, Glu, often contacting the terminal nitrogen of PE), and several types of neutral polar residues (Asn, Gln, Ser), as well as some contribution from the residues with hydrophobic side chains (Leu, Ala, Phe). For oxy-Mb, the pattern was much less clear, with heterogeneous but small contributions distributed among various amino acid types (Table S2).

In contrast to the average-composition OMM, at the contact-sites where oxy-Mb showed a very strong attachment and required almost double the force than deoxy-Mb, the oxy-Mb had a 2–3 times higher frequency of atomic contact with the lipids (Table S2), especially with PC and PE. Furthermore, the presence of cardiolipin (CL) at the contact sites, which was found at significantly higher concentrations, contributed to approximately one-third of the contacts with oxy-Mb. The strong correlation (0.93) between CL and the detachment force highlighted its critical role in the attachment of oxy-Mb to the contact sites, while playing a relatively minor role in stabilizing deoxy-Mb. The charged protein residues, both basic and acidic, contributed the most to both types of Mb, with Lys, Asp and, Glu (for oxy-Mb) being the most prominent. The second tier of contributions was from a few types of polar residues (Ser, Gln, Gly, His), with a small addition from nonpolar Leu (oxy-Mb).

The differences in the binding strength and preferred location between oxy- and deoxy-Mb suggest that as the O_2_ saturation waxes and wanes during the normal functioning of Mb-expressing cells such as cardiomyocytes, skeletal muscle cells, and brown adipocytes, the oxy-Mb and deoxy-Mb forms will toggle back and forth in abundance. This may lead to dynamic Mb–mitochondrial binding patterns, and redistribution between the contact and non-contact regions of the OMM. Further, it is anticipated that there may be membrane-induced conformational changes in oxy-Mb during interaction with the CS-OMM, especially with the longer ‘residence time’ at the interaction site. These membrane-induced conformational changes in oxy-Mb may trigger O_2_ release near the CS-OMM, and transition into deoxygenated forms. This could potentially facilitate the temporary dissociation of deoxy-Mb from the CS-OMM, which needs further investigation. We further hypothesize that after O_2_ release, the dissociation of deoxy-Mb from the CS-OMM may involve the diffusion of deoxy-Mb into the surrounding environment, or reattachment to the non-contact sites of the OMM, awaiting the acquisition of additional O_2_ from the cytosol. After the O_2_ re-binding, oxy-Mb may relocate back to the next CS-OMM, and toward O_2_ release. The present manuscript exclusively focused on investigating the specific orientation of oxy-Mb and deoxy-Mb with the OMM and their respective CS-OMM.

### 3.8. Regions of the Protein Stabilizing Contact with the Membrane in the Steered Detachment

**Figure 6:**
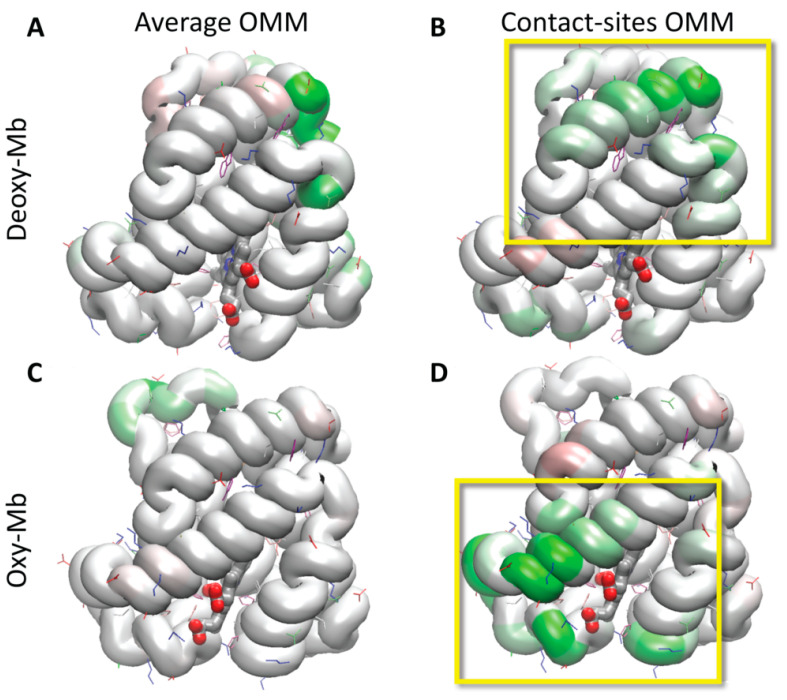
Maps of the contribution of the protein regions to the maximum force required for the steered detachment of mouse Mb from the average OMM (**A**) and (**C**) and the contact site OMM (**B**) and (**D**). The Mb protein is color-coded, ranging from red (residues that when present, contributed to destabilizing the contact) to green (residues contributing to stabilizing the interaction). The yellow frames highlight the difference between the most stabilizing regions for the adhesion of Mb to the contact site OMM, i.e., the N-terminal helix and nearby loop in deoxy-Mb, and the region surrounding the heme in oxy-Mb.

The contribution of the residues toward the force required for the steered detachment of Mb from the mitochondrial outer membrane was quantified as the product of frequency of the specific residue contact with lipids, and the maximum force for the whole protein required for separation. The reason for this evaluation was that the contacts between individual residues and the lipids varied significantly during the detachment, and often were cooperative or inter-dependent because both polar and hydrophobic contacts were involved, as well as interactions mediated by water and ions. This scaling approach allowed us to downplay the residues that were involved in strong attachments but only for a short period of time, as well as those residues that were contacting the OMM often, but with unstable attachments. As shown in Figure 6, the green colored regions map the regions that contributed the most to the stability of the contact. The maps show that the corner loops and N-terminal helix contributed significantly toward the energy required for the detachment of deoxy-Mb. In contrast, for the oxy-Mb at the contact sites, the most significant contribution to the detachment force came from the region close to the heme crevice of the protein.

### 3.9. Typical Orientation of the Bound Myoglobin Relative to the Membrane

**Figure 7:**
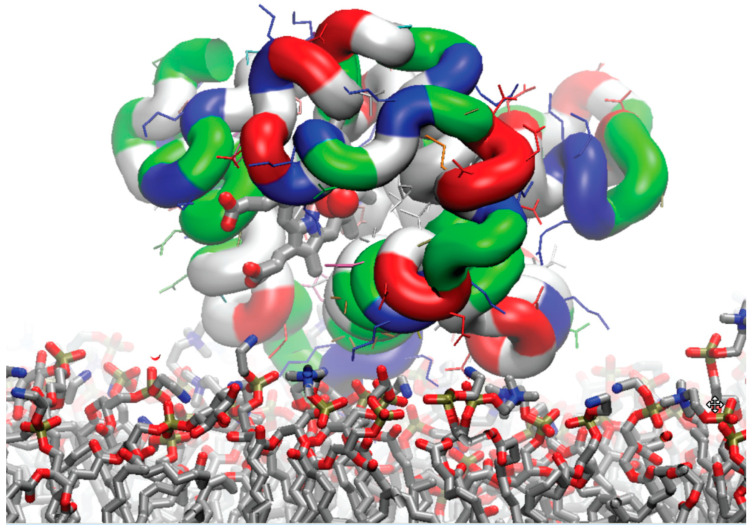
Representative configurations of the strongest oxy-Mb attachment at the contact sites of the OMM. Based on the conformations requiring the highest force for steered 30 ns detachments, the regions framing the heme cavity contribute the most, while the heme approaches close to the membrane, leaving only a short, solvated gap for the likely pathway for O_2_ release, thus facilitating the delivery. The protein is colored according to the residue type (nonpolar, white; polar neutral, green; basic, blue; and acidic, red.

Mb shows a pronounced variability in orientations and contacting residues when it binds to the OMM. However, according to the mapping, there are several regions that contribute the most to the stability of the attachment. For unrestrained relaxations, the frequently contacting regions include the loops at the corners of Mb, as well as the N-terminal helix, thus leaving a considerable solution-filled gap between the heme crevice and membrane. However, unrestrained simulations provide only a limited insight into the long-term stability of the contact. To illustrate Mb–membrane attachment configuration in the context of the potential routes of the O_2_ release on a longer time scale, we have selected a typical arrangement based on the analysis of steered simulations of oxy-Mb detachment from the contact-site OMM. The residues that were involved in the simulations with a higher force were located around the heme-containing crevice. The arrangement presented in Figure 7 shows a snapshot at the beginning of the steered detachment that required the highest force to separate protein from the membrane, thus suggesting a potential long residence time that might be difficult to estimate in unrestrained simulations. In agreement with the analysis of contributing residues, both the basic and acidic residues mediated the contact, with some contribution from the neutral polar residues. In such configurations, the heme crevice closely approached the membrane, creating a narrow-hydrated gap through which O_2_ could cross following its potential release from Mb. This arrangement effectively facilitated the offloading of O_2_ at the OMM.

## 4. Conclusions

In the current study, we used MD simulations with a heterogeneous outer mitochondrial membrane to predict interactions with deoxy- and oxy-Mb. By conducting simulations with multiple starting orientations of Mb, we were able to establish contact with the membrane using a small constant force bias. This approach enabled us to estimate the stability of the contact during subsequent unrestrained relaxations. The results showed high variability in the initial configuration of the protein–membrane contact, with the preference for the attachment to be mediated by the “corners” and the N-terminal helix of Mb. Deoxy-Mb showed a somewhat more stable attachment to the average OMM in unrestrained simulations, compared to oxy-Mb. Both deoxy- and oxy-Mb were more stable when attaching to the contact sites of the OMM than when attaching to the average-composition lipid patches, and this appeared to be stronger for oxy-Mb vs. deoxy-Mb. The similarity observed in the contact maps across different simulations, along with the comparison with the electrostatic 3D maps of the protein indicated that the geometry and stability of the initial contact arrangements were primarily determined by the protein’s geometry, and the transient local interactions between the partial charges on the protein and lipid residues. This suggested that these factors played a more significant role in shaping the contact arrangements than long-range electrostatic interactions.

When the force required for the detachment of bound Mb from the membrane was tested, it was discovered that deoxy-Mb needed a stronger force to leave the average-composition OMM than it did to leave the contact-site OMM. In contrast, oxy-Mb showed a clearly stronger attachment at the contact-site OMM. The analysis of the contributions from different lipid types showed that the attachment at the average-composition sites, especially for deoxy-Mb, was stabilized by interactions with the PC and PE headgroups. In contrast, the binding of oxy-Mb at the contact sites of the OMM showed a large stabilizing contribution from the charged lipids, especially CL. The spatial distribution of the protein residues that contributed to stabilizing the attachment of oxy-Mb to the contact sites on the OMM suggested that the most stable arrangements occurred when Mb positioned its heme crevice toward the membrane. This positioning created a narrow layer of solute, facilitating the diffusion of O_2_ during its potential release from Mb. Such arrangements are expected to enhance the effective delivery of O_2_.

These results highlight that, as Mb converts back and forth between the oxy- and deoxy- forms, which takes place with natural ebbs and flows in pO_2_ or, in the extreme, with hypoxia/ischemia, the contact dynamics of Mb to the OMM of mitochondria change. An intriguing finding was the strong binding of oxy-Mb observed near contact sites in the OMM. This observation suggests that the protein could be located in close proximity to the electron transport chain complex. Subsequent investigations should prioritize exploring the functional implications of Mb–OMM contact and dissociation. Additionally, it would be intriguing to investigate whether the proximity of Mb to the OMM enhances its binding affinity with metabolites such as lipids, lactate, or pyruvate. Furthermore, studying the potential influence of Mb–metabolite binding on the strength, location, or O_2_ offloading at the Mb–OMM interface would provide valuable insights.

Additionally, to unravel the intricate details of the conformational changes and Mb-mediated O_2_ release, future studies employing a comprehensive approach, integrating MD simulation coupled with quantum mechanics experiments, are needed. In this direction, we strive to develop a quantitative model that accurately represents the essential steps in O_2_ migration and rebinding, with a particular emphasis on the O_2_–heme binding/unbinding reaction in Mb. This approach will also provide valuable insights into the distinct pathways and intermediate binding sites involved in the transport and exchange of O_2_ molecules from Mb. In addition to the crucial role of Mb in facilitating O_2_ transport, it is also important to recognize the potential impact of O_2_ detachment from the heme group, and its subsequent diffusion into the mitochondrial membrane. The membrane-induced conformational changes in Mb may lead to the reorganization of its internal cavities and hold significant implications for the overall functionality of Mb. These investigations will not only evaluate the sequential transitions between specific cavities and channels within the Mb structure, but also explore the efficient binding and release of O_2_ from Mb when bound to the OMM. Moreover, the critical residues and structural features that contribute to the stability and selectivity of O_2_ migration will be identified. We anticipate that these comprehensive investigations will significantly enhance our knowledge and understanding of the intricate mechanism underlying Mb–O_2_ transport. Furthermore, it will establish a solid foundation for further exploration toward the physiological significance and potential applications of Mb in O_2_ homeostasis and cellular metabolism.

## Figures and Tables

**Table 1:**
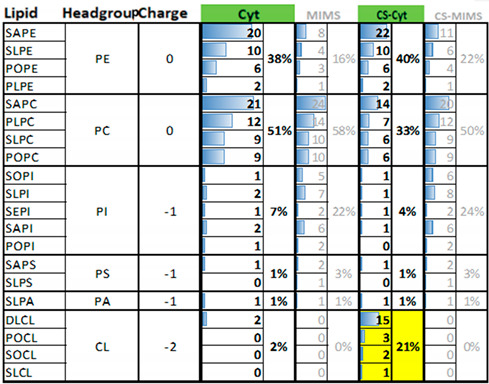
The lipid composition of the membrane patches, assembled to reflect the average fractions of the headgroups and lipid tails of the outer mitochondrial membrane (OMM), separately for the whole-membrane average, and for the contact sites of the outer membrane with the cristae (CS-OMM). The external surface of the CS-OMM sites is characterized by a higher concentration of cardiolipins (highlighted in yellow). Columns Cyt, MIMS, CS-Cyt, and CS-MIMS show both the number of the specific lipid type (the left part with the histograms), and the total percentage of the given headgroup type relative to the whole monolayer.

Abbreviations: Cyt, cytoplasmic side of the mitochondrial outer membrane; MIMS, mitochondrial intermembrane space side of the membrane; CS-Cyt, contact sites of the cytoplasm-facing side of the membrane; CS-MIMS, contact sites of the mitochondrial intermembrane space side of the membrane; SAPE, stearoyl arachidonoyl phosphatidyl ethanolamine; SLPE, stearoyl linoleoyl phosphatidyl ethanolamine; POPE, palmitoyl oleoyl phosphatidyl ethanolamine; PLPE, palmitoyl linoleoyl phosphatidyl ethanolamine; SAPC, stearoyl arachidonoyl phosphatidyl choline; PLPC, palmitoyl linoleoyl phosphatidyl choline; SLPC, stearoyl linoleoyl phosphatidyl choline; POPC, palmitoyl oleoyl phosphatidyl choline; SOPI, stearoyl oleoyl phosphatidylinositol; SLPI, stearoyl linoleoyl phosphatidyl inositol; SEPI, stearoyl eicosatrienoyl phosphatidyl inositol; SAPI, stearoyl arachidonoyl phosphatidyl inositol; POPI, palmitoyl oleoyl phosphatidyl inositol; SAPS, stearoyl arachidonoyl phosphatidyl serine; SLPS, stearoyl linoleoyl phosphatidyl serine; SLPA, stearoyl linoleoyl phosphatidic acid; DLCL, eicosadienoyl linoleoyl cardiolipin; POCL, palmitoyl oleoyl cardiolipin; SOCL, stearoyl oleoyl cardiolipin; SLCL, stearoyl linoleoyl cardiolipin; PE, phosphatidyl ethanolamine; PC, phosphatidyl choline; PI, phosphatidyl inositol; PS, phosphatidyl serine; PA, phosphatidic acid; CL, cardiolipin.

**Table 2:** The minimum observed number of contacts between the heavy atoms of Mb and the OMM during the 40 ns relaxation. Each simulation cell represents an independent Mb–membrane attachment (12 per Mb–membrane-type combinations, see Methods for details). The number of contacts was tracked throughout the simulation, with a 5 ps time step. The values in the table represent the lowest number of contacts that was observed at any point for the given attachment. For easier visual comparison, the cells are sorted and color-coded, to denote the range from the largest (green) to the lowest (red) values, regardless of the specific order of the simulations.

	Average OMM		Contact-Sites OMM	
Independent Runs	Deoxy-Mb	Oxy-Mb	Deoxy-Mb	Oxy-Mb
	39	15	56	26
	38	12	41	21
	27	8	30	18
	17	5	29	10
	9	3	20	4
	0	2	5	2
	0	0	3	1
	0	0	2	0
	0	0	1	0
	0	0	0	0
	0	0	0	0
	0	0	0	0
**Average**	**10.8**	**3.8**	**15.6**	**6.8**
**CS/Avg OMM Ratio**			**1.4**	**1.8**
**Oxy/Deoxy Mb Ratio**		**0.3**		**0.4**

**Table 3:** The average force required to detach Mb from the membrane. The force was measured using 30 ns steered detachment simulations, with the constant velocity movement of the harmonically restrained center-of-mass of the protein. Each cell represents an individual Mb molecule, color coded to denote the range from the smallest (red) to the medium (yellow) to the highest (green) force required for detachment. The empty cells represent simulations where there was no significant initial contact (lost during the previous relaxation simulations).

	Average OMM		Contact-Sites OMM	
Independent Runs	Deoxy-Mb	Oxy-Mb	Deoxy-Mb	Oxy-Mb
	150.92	62.13	57.65	126.39
	80.16	45.48	53.54	102.9
	72.58	43.94	46.74	97.19
	57.09	39.07	46.73	93.11
	51.98	27.55	45.88	88.53
	40.8	27.29	41.49	65
	40.11	25.98	25.76	60.72
	37.36	24.83	16.65	38.61
	30.14	14.08	13.01	28.79
	18.49	13.36	12.55	24.89
	12.89			7.99
	9.67			
**Average**	**50.2**	**32.4**	**36.0**	**66.7**
**CS/Avg OMM Ratio**			**0.7**	**2.1**
**Oxy/Deoxy Mb Ratio**		**0.6**		**1.9**

## Data Availability

Not applicable.

## Supplementary Materials

The following supporting information can be downloaded at: https://www.mdpi.com/article/10.3390/biom13071138/s1, Figure S1: Six “sides of the cube” orthogonal starting orientations that were used for both oxy- and deoxy-Mb simulations of associations with the OMM. The mouse Mb protein in this representation is colored by the residue number (N-terminus red, C-terminus blue). The heme is in licorice representation (yellow), while the lipid membrane surface shown as “sticks” colored by the atom type (carbon—gray, oxygen—red, nitrogen—blue, and phosphorus—tan); Figure S2: Illustration of the freedom of systems to adjust, and variability of the contact orientations of mouse Mb to the OMM, during simulations of Mb-mitochondrial membrane associations. Top panel (A): Change in the lipid’s arrangement during 40 ns establishment of the contact between Mb and the membrane patch at the contact-site OMM. Lipids are randomly colored (the same color on both panels) to illustrate the lateral diffusion on the scale of up to 3 nm that leads to the significant changes in the lipid arrangement and transient clustering. A similar range of diffusion occurs at the average OMM. Lower panel (B): Five simulations of constant-force attachment of oxy-Mb to the membrane, all started with identical initial coordinates (but having some randomized component rue to the Langevin dynamics used for temperature and pressure control), produce distinct outputs. The rotation of the protein can vary on the scale of about 90 degrees from the initial orientation, while lateral diffusion is in the range of 1 nm. Deoxy-Mb shows comparable range of variation in orientations at the same conditions; Figure S3: Examples of two predominant kinds of Mb-mitochondrial membrane attachment stability dynamics typically observed in the course of unrestrained 40 ns relaxation simulations, shown here for oxy-Mb at the contact-site OMM. (A, left panel) A stable attachment where neither the average number of atomic contacts nor the average distance from the membrane plane change significantly within the simulation time but rather just fluctuate around the same values; z coordinate shows the position of Mb center of mass, in this case negative for the Mb at the “lower” monolayer. (B, right panel) an unstable attachment where the total number of Mb-membrane contacts quickly decreases from 85 down to 0-1, whereas center of mass of Mb (contacting the “upper” monolayer) moves away from the membrane by up to 20 Å. The traces are color-coded according to the simulation time, from start (red) to end (blue); Figure S4: Examples of a stable and unstable Mb-mitochondrial membrane attachments. The left column represents oxy-Mb attached to the contact sites of the OMM at the beginning of 40 ns relaxation, while the right column represents contact characteristics at the end of the relaxation period. The top row shows one of the stable attachments, and the bottom row depicts one of the unstable arrangements. These results are a subset of the molecular modeling analysis runs in which mouse Mb was placed near the OMM in six different orientations to start, to determine which parts of the Mb most frequently bind with stability; Table S1: The stability score of the Mb contacts with the membrane for unrestrained 40 ns simulations after the initial contact was established. The “stability score” of an individual Mb-membrane attachment was quantified as the ratio of (1 + the lowest seen number of contacts) / (the largest seen number of contacts). Each cell in the table represents an individual Mb molecule. The table is color-coded from the least stable (red), to intermediate (yellow), to the most stable (green) arrangements; Table S2: Contribution the amino acid and lipid types to the stability of mouse Mb-mitochondrial membrane contact in steered detachment simulations for the (A) average OMM and (B) contact-sites OMM. The average contact frequency was estimated as time-averaged number of contacts for heavy atoms for the residue of a particular type. The contact frequency in the table is color-coded from white (the lowest number of contacts) to green (the highest). Correlation of particular residue type contacts with the average force required for detachment was calculated based on the whole set of simulations for the given system type (e.g., deoxy-Mb with the contact sites of OMM), up to 12 independent simulated detachments as described in Methods. Correlation cells are color-coded from red (negative correlation) to blue (positive). The importance score is a product of the correlation with the energy and the average frequency of contacts and color-coded from white to burnt orange.

## Abbreviations

OMM, outer mitochondrial membrane; CS-OMM, contact site outer mitochondrial membrane; deoxy-Mb, deoxygenated myoglobin; Mb, myoglobin; oxy-Mb, oxygenated myoglobin.
