# Supplementary figures and images for: Computational Analysis Reveals Unique Binding Patterns of Oxygenated and Deoxygenated Myoglobin to the Outer Mitochondrial Membrane
Source: Biomolecules. 2023 Jul 17;13(7):1138. doi: 10.3390/biom13071138 (PMC10377724; doi:10.3390/biom13071138)

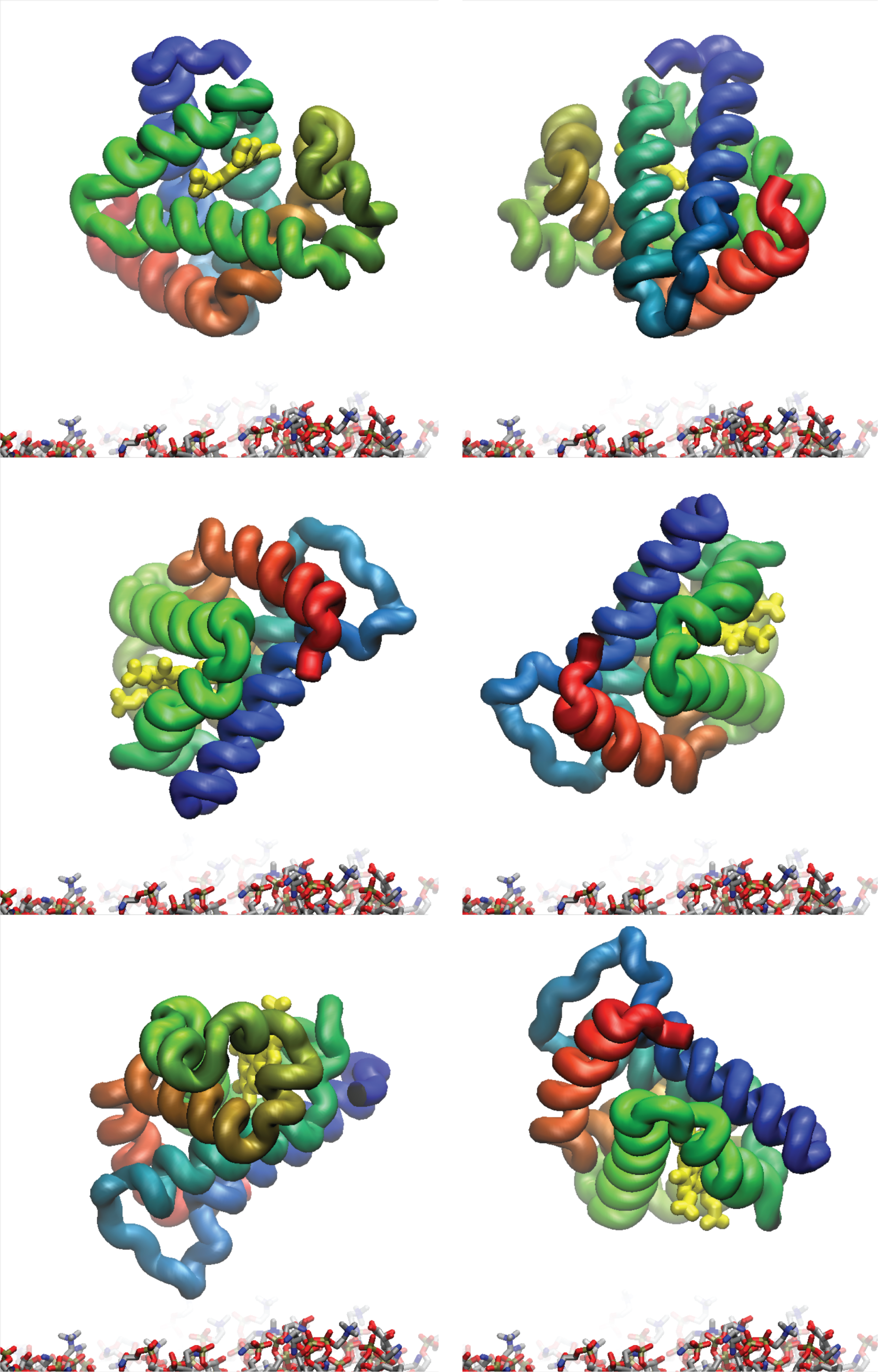

Supplement: Supplementary file 1 [file biomolecules-13-01138-s001.zip › Figure. S1.tif]

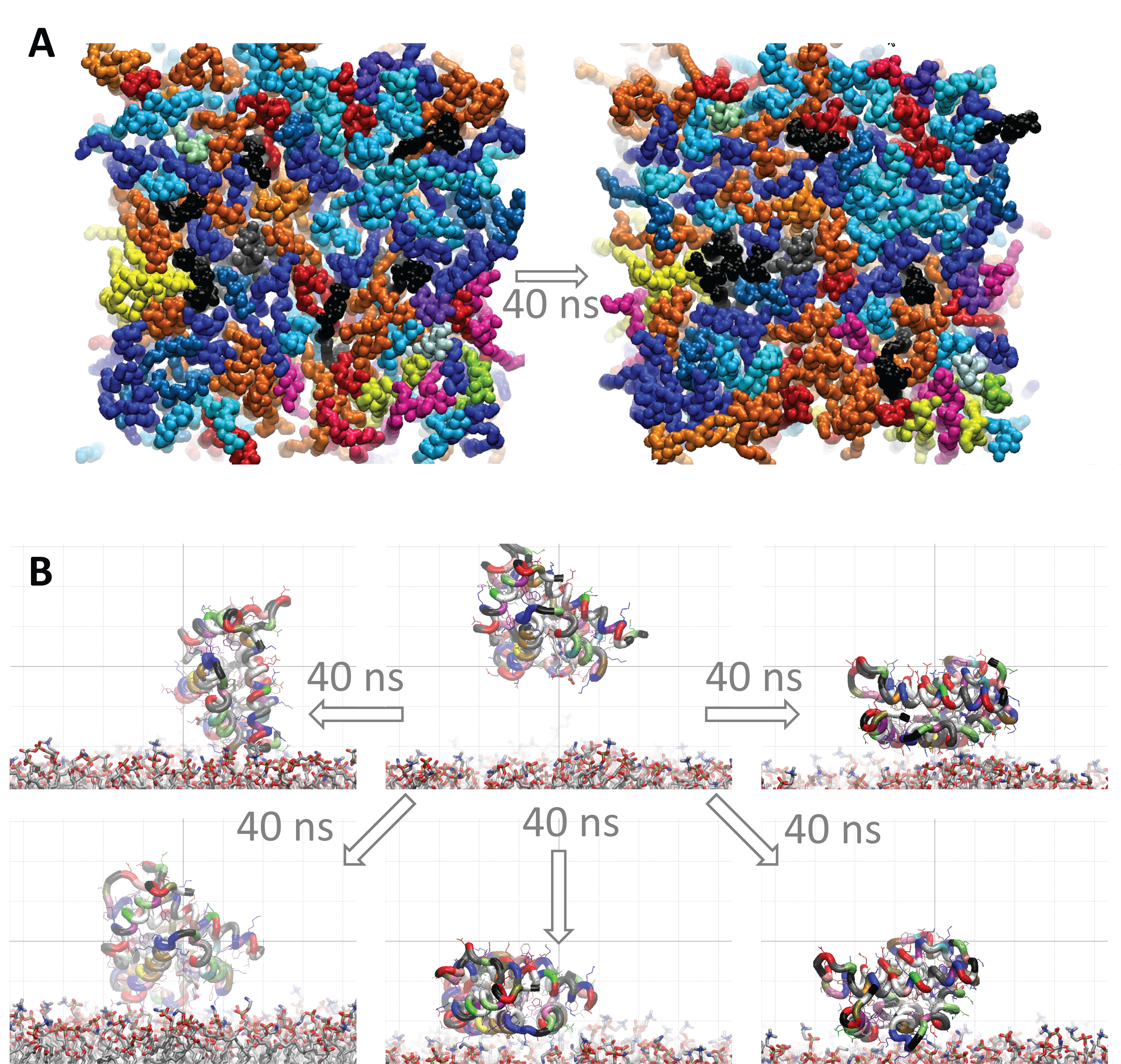

Supplement: Supplementary file 1 [file biomolecules-13-01138-s001.zip › Figure. S2.tif]

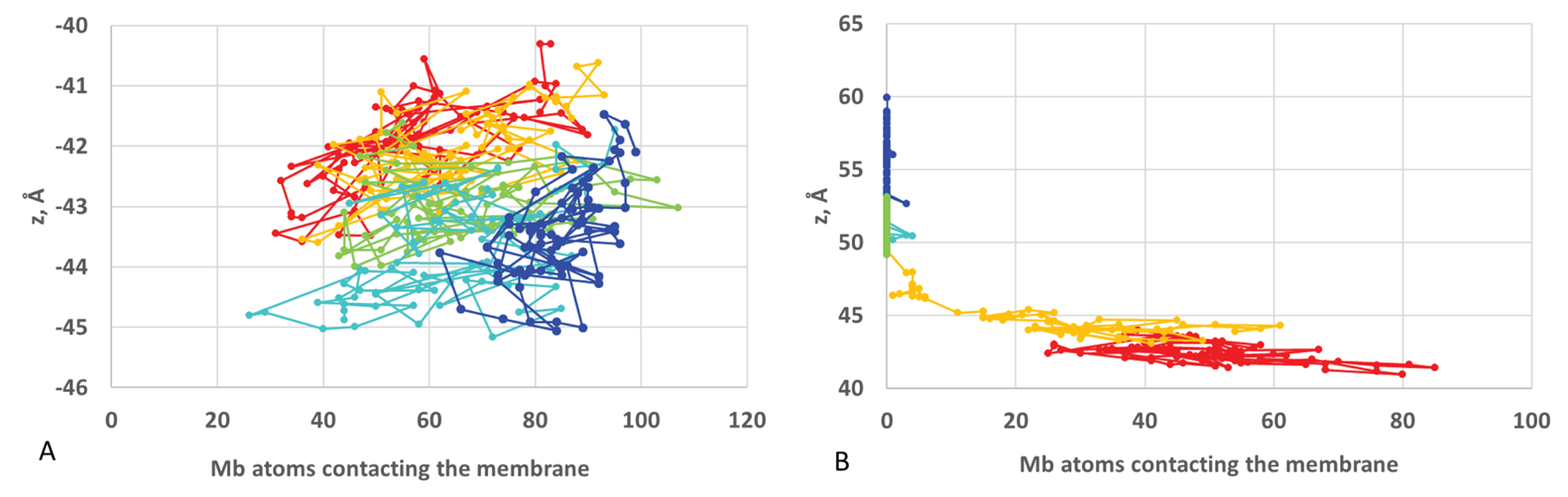

Supplement: Supplementary file 1 [file biomolecules-13-01138-s001.zip › Figure. S3.tif]

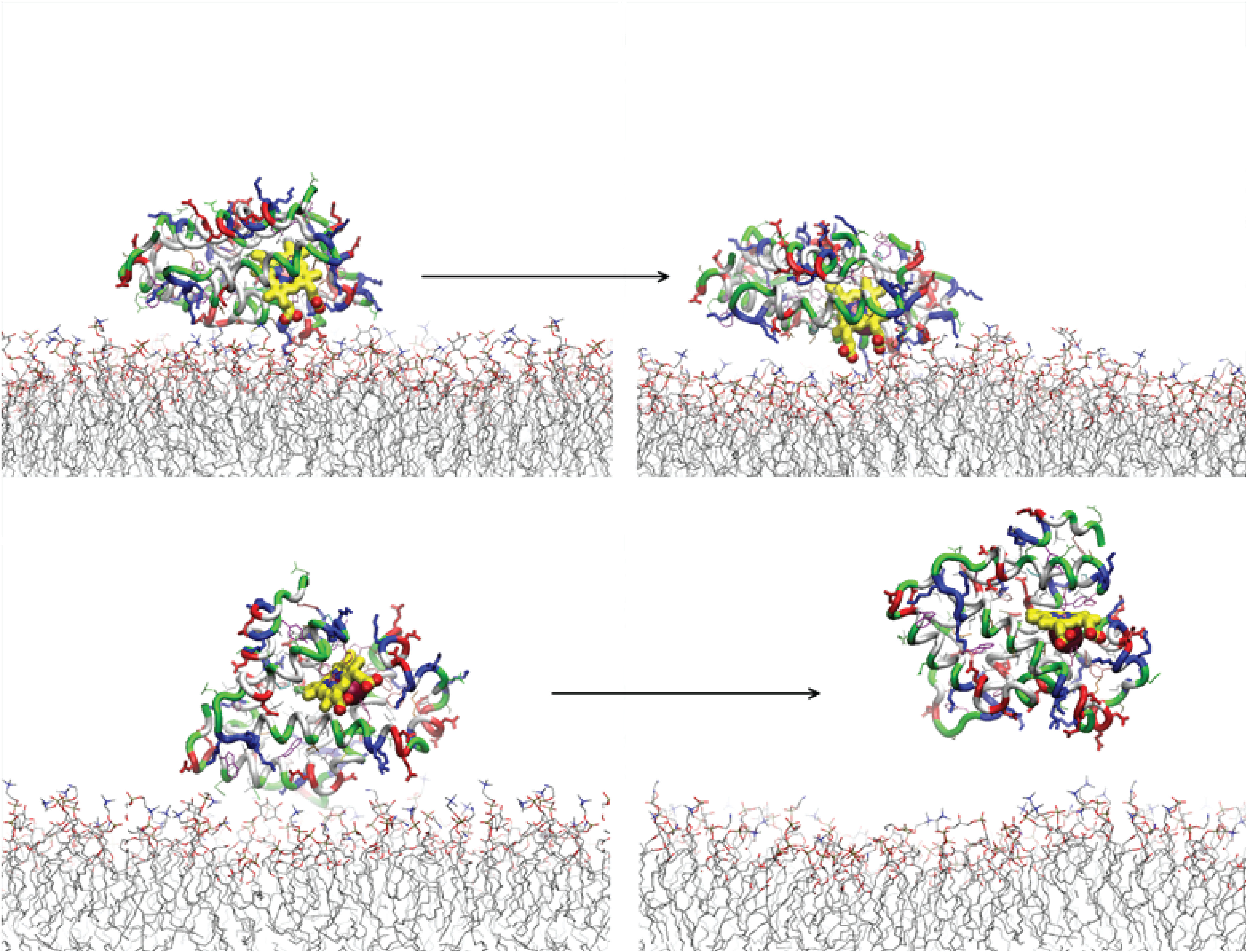

Supplement: Supplementary file 1 [file biomolecules-13-01138-s001.zip › Figure. S4.tif]

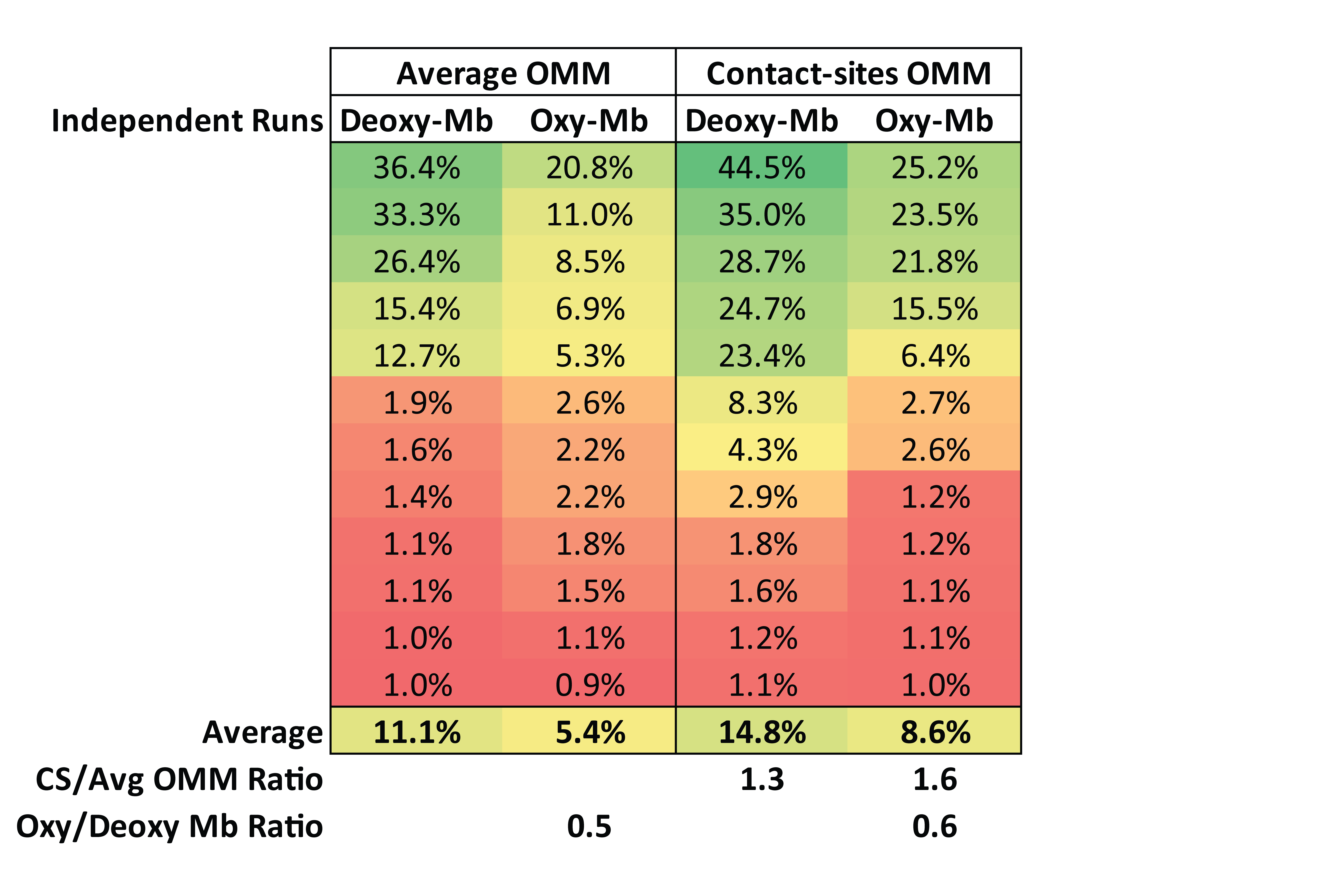

Supplement: Supplementary file 1 [file biomolecules-13-01138-s001.zip › Table S1.tif]

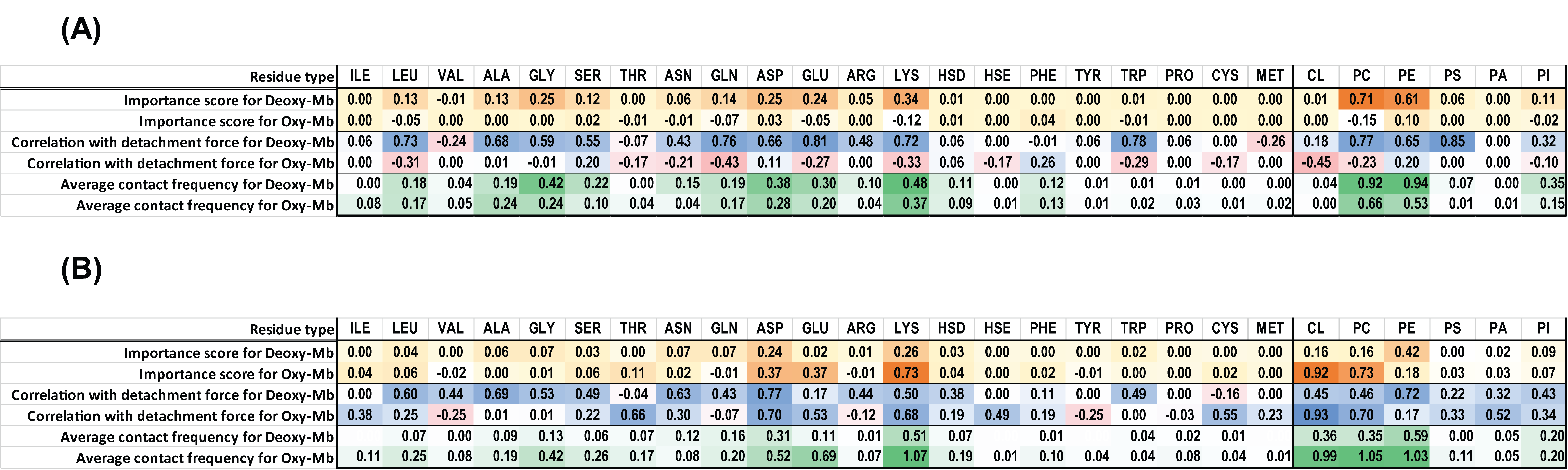

Supplement: Supplementary file 1 [file biomolecules-13-01138-s001.zip › Table S2.tif]
